# Cross-Cultural Adaptation and Validation of the Beck Depression Inventory (BDI-II) in the Community Otomi of the Mezquital Valley, Mexico

**DOI:** 10.3390/healthcare13243326

**Published:** 2025-12-18

**Authors:** Irene López-Hernández, Claudia Lerma, Rebeca María Elena Guzmán-Saldaña, Itzel Moreno Vite, María Luisa Escamilla Gutiérrez, Cristina J. González-Flores, Abel Lerma

**Affiliations:** 1 Institute of Health Sciences, Universidad Autónoma del Estado de Hidalgo, San Juan Tilcuautla 42160, Mexico; lo263737@uaeh.edu.mx (I.L.-H.); rguzman@uaeh.edu.mx (R.M.E.G.-S.); itzel_moreno@uaeh.edu.mx (I.M.V.); maria_escamilla@uaeh.edu.mx (M.L.E.G.); 2 Departamento de Biología Molecular, Instituto Nacional de Cardiología Ignacio Chávez, Mexico City 04480, Mexico; 3 Centro de Investigación en Ciencias de la Salud (CICSA), Facultad de Ciencias de la Salud, Universidad Anáhuac México, Huixquilucan 52786, Mexico; 4 Centro Universitario de la Cienega, University of Guadalajara, Ocotlán 47820, Mexico; cristina.gonzalez4243@academicos.udg.mx

**Keywords:** depressive symptoms, indigenous, ethnic minority mental health, cross-cultural psychology, mental health disparities

## Abstract

**Background:** The Beck Depression Inventory Second Edition (BDI-II) is used to assess depression worldwide. In Mexico, the BDI-II Spanish translation is widely used. Despite more than 23 million people being identified as indigenous, there is no empirical evidence on the BDI-II psychometric properties among indigenous languages, including Otomi. Therefore, this study aimed to cross-culturally adapt the BDI-II for the Otomi population and evaluate its psychometric properties. **Methods:** This cross-sectional instrumental study with non-probability sampling was conducted with 228 participants from the Otomi community. The cross-cultural adaptation of the BDI-II followed Beaton’s guidelines for self-report measures: (i) translation, (ii) synthesis, (iii) back translation, (iv) expert committee review, (v) pretesting, and (vi) submission of documentation to the developers. Reliability was assessed using Cronbach’s alpha. Exploratory and confirmatory factor analyses were used to determine structural and construct validity. **Results:** The cross-culturally adapted instrument showed adequate reliability, with a total Cronbach’s α of 0.756, comprising 14 items and four factors (with alpha coefficients ranging from 0.505 to 0.633). These factors included three cognitive–affective dimensions and one somatic dimension, which conceptually align with Beck’s original model. Confirmatory factor analysis (CFA) presented adequate indices: Comparative Fit Index (CFI) = 0.901, Root Mean Square Error of Approximation (RMSEA) = 0.056, IC_90%_ [0.028–0.079], and Goodness-of-Fit Index = 0.908, which indicate a balanced and parsimonious fit of the model. **Conclusions:** The BDI-II is a reliable and culturally valid instrument for measuring depressive symptoms among the Otomi people of the Mezquital Valley.

## 1. Introduction

Depression is the leading cause of disability worldwide, affecting 4% of the population, with a prevalence of 5.7% in adults [1]. This clinically significant disorder is among the most prevalent psychiatric conditions globally [2]. It occurs more frequently in women, and indigenous populations have up to five times higher risk of experiencing it [3]. In Mexico, the depression prevalence rose to 31.1% in adolescents and 16.7% in adults in 2022 [4]. Furthermore, the prevalence of depressive symptoms in adults aged 20 to 59 years varies according to the indigenous group and literacy level [5]. The BDI-II is the most widely used instrument for measuring the frequency and intensity of depressive symptoms. This instrument has proven useful and has acceptable psychometric properties worldwide, both in the general population [6,7,8,9,10] and in clinical populations [11,12,13,14,15,16]. The BDI-II has been validated in the general Mexican population [17,18,19] and in a Mexican clinical population [20,21,22], demonstrating adequate internal consistency and factorial structure.

In Mexico, 23.2 million people aged three and older identify as Indigenous, and 7.4 million of them speak an Indigenous language, of whom 866,000 do not speak Spanish [23]. The Mezquital Valley is located in the central part of the state of Hidalgo, in the central highlands of Mexico. It is bordered to the west by the large valleys of the Bajío, to the south by the Valley of Mexico, and to the east and north by the Sierra Madre Oriental [24]. Sociocultural factors define the region’s territorial limits: language, food, housing, and social organization. It comprises twenty-seven municipalities [25]. Ixmiquilpan, often referred to as the heart of the Mezquital Valley, is one of the ten geographical regions in the state of Hidalgo [26]. This is a rural area characterized by social marginalization, with a community organization based in patriarchal household units [27]. Assembly and community affiliation are the basis for community organization and a strategy of daily resistance to strengthen its autonomy [28]. The high rate of migration has made it a key element of local development [29], leading to emotional expressions such as ndumu̱i (sadness) and beni (longing) among family members who stay behind in the community [27].

Having culturally adapted and valid psychometric tools is essential to detect depression in communities with limited access to mental health services [30]. The use of Western psychiatric models without considering the cultural context leads to hermeneutical injustice, misdiagnoses, and institutional distrust [31]. Conversely, language adjustments can increase the acceptance of psychometric tools without compromising their validity [32]. Moreover, empirical adaptation studies are necessary because the expression of depression varies across cultures and languages [33]. Some of the instruments used to measure depression in indigenous communities include the Patient Health Questionnaire (PHQ-9), which has been validated in three variants of Quechua in Perú [34]; the short version of the Center for Epidemiological Studies Depression Scale (CES-D) was used in indigenous populations in Mexico [5]; and in Ecuador, the BDI was translated and culturally adapted into Kichwa Cañari [35]. Beck’s original model identified a bifactorial structure [36] and a trifactorial structure [37]. However, empirical studies have shown discrepancies in the factorial structure of the BDI-II compared to the original model. Some studies have replicated the original factorial structure [22,35], while others have proposed a unifactorial model [16], a bifactorial model [18,19,20,38], or a more complex trifactorial structure [6,11,12,13,17,39].

Although the BDI-II has been validated in various populations and cultures worldwide, in Mexico, there is a lack of empirical evidence regarding its factorial structure among the indigenous Otomi people, particularly in the linguistic variant “Otomi of the Mezquital Valley,” for evaluating depression in this specific social context. Therefore, the objective of this study was to cross-culturally adapt and analyze the psychometric properties of the Beck Depression Inventory (BDI-II) for the Otomi general population of the Mezquital Valley. Three specific objectives were posed: (1) to determine the reliability of the instrument, (2) to determine the factorial structure through exploratory factor analysis, and (3) to validate the factorial structure through confirmatory factor analysis. A two-factor [36] (cognitive–affective and somatic) or three-factor [37] (cognitive, affective, and somatic) structure was expected, as well as acceptable reliability of the scale and its factors.

## 2. Materials and Methods

### 2.1. Study Design and Participants

A total of 228 residents of the Otomí community of the Mezquital Valley participated, selected through non-random sampling. The median age of participants was 51 (41–60), with 61% being women. The inclusion criteria were: speaking the Otomi language of the Mezquital Valley, even if they also spoke Spanish; being bilingual; self-identifying as Indigenous; being of both sexes; being 18 years of age or older; and signing an informed consent form. Individuals who reported severe mental illness (schizophrenia, bipolar disorder, psychotic disorders, or severe depression) were excluded. Those who provided incomplete responses were also eliminated.

The principal investigator contacted the participants directly at the central garden in the municipality of Ixmiquilpan, Hidalgo, located in front of the municipal government building. This space is frequented by residents of the municipality’s 119 localities. The instrument was administered individually, in printed format, from July to December 2024. The sample size was determined according to the most common rule, which is a ratio of 3 to 10 participants per item for factor analysis [40,41]. Similarly, other authors have suggested using between 5 and 10 participants per item [42,43].

### 2.2. Assessment Tools

Sociodemographic data questionnaire. A form was created to collect information on participants’ sociodemographic variables, including age, sex, educational level, marital status, chronic illness, health insurance, and attendance at psychological care.

Beck Depression Inventory, Second Edition (BDI-II) [36]. It consists of 21 items that assess symptoms of depression. Participants respond on a four-point scale (from 0 to 3), with higher scores reflecting severe depressive symptoms. The Spanish version of the BDI-II was used, which was validated in Mexican students and adults with a reliability of α = 0.87 in the adult population [17].

### 2.3. Cross-Cultural Adaptation and Validation Procedure

#### 2.3.1. First Stage: Cross-Cultural Adaptation

The cross-cultural adaptation of the BDI-II to the indigenous Otomí population of the Mezquital Valley was carried out following the guidelines for the cross-cultural adaptation process of self-report measures [44], which include the following: (i) initial translation, which consists of two independent versions: one formal and one informal, produced by two translators independently; (ii) synthesis discrepancies between translations are resolved; (iii) back translation consists of an inverse translation of the synthesis generated in the previous stage; (iv) review by a committee of three experts; (v) pretesting; and (vi) submission and evaluation of all written reports by the author. The three experts, who spoke Otomi, evaluated six criteria: relevance, writing, language appropriateness for the population, theoretical validity, appearance validity, and content validity. Then, we performed a content validity analysis [45]. Before pretesting, a preliminary survey was conducted as a face validity test with three people to clarify meanings and identify any unknown words. Pretesting involves administering the instrument to a sample of 30 participants from the target population to ensure a thorough understanding of all items. All participants in the pretest demonstrated a clear understanding of the instrument’s content, with only suggestions to improve the format (larger font, better column separation). The final version of the cross-cultural adaptation of the BDI-II for the Otomi population of the Mezquital Valley was obtained.

#### 2.3.2. Second Stage: Psychometric Properties of the Instrument

The final version of the BDI-II, cross-culturally adapted for the Otomi population of the Mezquital Valley, was administered individually as a printed version. Participation was voluntary, as expressed by signing an informed consent form. All participants were informed of their rights, as well as the objectives and scope of the research. They were also provided with precise instructions on how to complete the instrument. During the application, any questions related to the content, vocabulary, and completion of the instrument were addressed. The person who applied the instrument identifies himself as Otomi and speaks the Otomí language of the Mezquital Valley. The procedures for data collection were rigorously followed, and subsequently, independent analyses were performed by two members of the research team to reduce bias in the results.

### 2.4. Statistical Analysis

#### 2.4.1. Descriptive and Inferential Analysis

Data were analyzed using the Statistical Package for the Social Sciences (SPSS) version 21.0 for Windows. The normal distribution of quantitative variables was assessed using the Kolmogorov–Smirnov test; results are reported as mean ± standard deviation or median (interquartile range: 25th percentile–75th percentile).

Inspired by the cut-off points defined for depression symptom severity in the BDI-II validated in Mexico, the total depression symptoms were calculated from the final validated version, and quartiles 1 to 4 were assessed as potential empirical threshold values of depressive symptom severity as follows: minimum (or normal) symptoms < percentile 25; percentile 25 ≤ mild symptoms < percentile 50; percentile 50 ≤ moderate symptoms < percentile 7; and severe symptoms ≥ percentile 75. Then, the participants’ characteristics were compared between those with minimum (normal) symptoms and those with mild or higher severity using the Student *t*-test or the Mann–Whitney U test.

#### 2.4.2. Exploratory Factor Analysis

A descriptive analysis was performed for each item to identify outliers and invalid frequencies. Subsequently, a variable was created by summing the 21 items. Based on the extreme quartiles 1 and 3, a Student *t*-test for independent samples was performed to assess the discriminatory capacity of the construct measured. The directionality of the items was verified through cross-item analysis. The correlation between items was evaluated to assess their magnitude, directionality, and significance, in order to determine the type of factor analysis to use. Cronbach’s alpha, the inter-item correlation, and the item-total correlation were calculated, verifying that no item had an increased alpha.

Regarding the first specific objective (to determine the scale’s reliability), the internal consistency of the factors and the total scale was assessed using Cronbach’s alpha coefficient. The following interpretation criteria were considered: α ≥ 0.70 as acceptable, α ≥ 0.80 as good, and α ≥ 0.90 as excellent [46]. These calculations were performed with a total sample of 228 participants.

Regarding the second objective (to determine the factor structure), an exploratory factor analysis (EFA) with Varimax rotation was performed, as the inter-item correlations ranged from low to medium, with a significance level of 95%. The rotated component matrix was analyzed to identify items loading on multiple factors. The factor structure’s usefulness is assessed using Bartlett’s sphericity test (*p* ≤ 0.05) and the Kaiser–Meyer–Olkin (KMO) index to evaluate the sample’s adequacy.

#### 2.4.3. Confirmatory Factor Analysis

Regarding the third objective (validating the factor structure through a confirmatory factor analysis with the maximum likelihood method in AMOS v.23), the fit of the 4-factor model was evaluated using the factor structure resulting from the EFA. This analysis was performed on a random sample of 60% (n = 138) using SPSS v21.0 for Windows, with simple random sampling from the original database (n = 228).

The following procedure was performed: (i) identification and specification of the model; (ii) estimation of standardized parameters: R2, modification indices, critical proportions of the difference, correlations, and covariances; and (iii) evaluation of the model fit by monitoring the indicators and ensuring the absence of collinearity between the observed variables [43,47].

The ratio between chi-square and the degrees of freedom X^2^/df (CMIN/DF) and X^2^ (CMIN) was estimated as a measure of model parsimony; the Goodness-of-Fit index (GFI), its adjusted version (AGFI), the Tucker–Lewis Index (TLI), the Comparative Fit Index (CFI), the Root Mean Square Error of Approximation (RMSEA), the Root Mean Square Residual (RMR), and the sampling adequacy index with the Holter test were estimated [48].

## 3. Results

### 3.1. Cross-Cultural Adaptation

The Spanish version of the BDI-II underwent a rigorous intercultural adaptation process tailored for the indigenous Otomi population of the Mezquital Valley, as described in Section 2.3.1. This process aimed to ensure both semantic and conceptual equivalence with the original version. The content validity analysis, based on evaluations by three experts in the Otomi language, selected for their linguistic and cultural competence to ensure the semantic and conceptual equivalence of the translation, is shown in Table 1. Table 2 presents the relevance proportion of the BDI-II, as determined from the 21 items. These results indicated that the translated questionnaire was equivalent to the original Spanish version, as assessed by the experts. The pilot test of the translated questionnaire revealed that the target population understood it, and the questionnaire did not require any further modifications.

### 3.2. Sociodemographic and Clinical Characteristics of Study Participants

The total study sample (n = 228) was composed predominantly of women, with a wide age range and diverse educational backgrounds (Table 3). The majority had not received psychological care or did not have health insurance. After inventory validation (as described below), the final, adapted, and validated questionnaire was obtained, which revealed the following quartiles for the total BDI-II score. The use of quartiles allows the observed distribution to be divided into four equivalent groups and to establish empirical thresholds that fit the characteristics of the population being assessed. In this study, the following cutoff points and depression levels are proposed: 0–8 = minimal depression, 9–13 = mild depression, 14–20 = moderate depression, and 21–42 = severe depression.

Considering the distribution by quartiles, participants located in quartiles 2, 3, and 4 were classified as having depression, while those in quartile 1 were considered without depression (Table 3). Compared to those without depressive symptoms, the group with depression had a higher proportion of women and a lower proportion of individuals with chronic illnesses. There were no significant differences between the groups in terms of sex, educational level, marital status, health insurance, or psychological care.

### 3.3. Exploratory Factor Analysis

Exploratory factor analysis using the principal components method and Varimax rotation, based on the scree plot and the rotated component matrix, yielded a structure of 14 items and four factors (with alpha coefficients of 0.505 and 0.633, respectively) with a total Cronbach’s alpha of α = 0.756, explaining 52.4% of the variance (Table 4).

**Figure 1:**
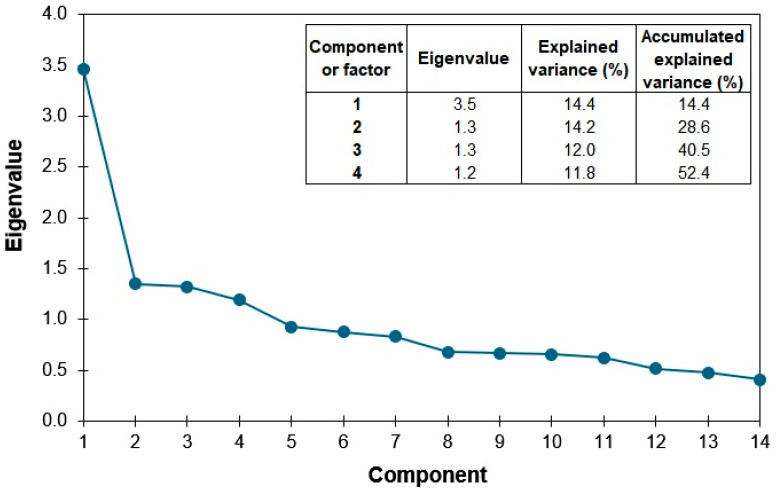
Scree plot of eigenvalues from the factor analysis of the Beck Depression Inventory (BDI-II) in the general Otomi population of the Mezquital Valley.

The number of factors to retain was determined using both the Kaiser criterion (eigenvalues > 1) and the scree plot (Figure 1), which shows the decline in eigenvalues for each factor. A clear inflection point is observed after the fourth factor, supporting the choice of a four-factor solution.

In this study, we propose naming the four factors as follows: F1: Cognitive-Affective 1; F2: Somatic; F3: Cognitive-Affective 2; F4: Cognitive-Affective 3, adhering to the reported factor names [36] and the similarity in the distribution of items. Validity estimates were also calculated for the total scale and for each of the factors (Table 4). Global scale α = 0.756 and explained variance = 52.4%. The Kaiser–Meyer–Olkin (KMO) test yielded a value of 0.768 (*p* = 0.001), confirming that the sample was adequate for the analysis. The remaining indices for the total sample of the 14 items were: total variance explained = 52.4%; mean = 9.91 ± 5.99; variance = 35.95; Hotelling’s T-square test = 644.26, *F* value = 46.94, *p* ≤ 0.001. Bartlett’s test of sphericity = approximate chi-square of 523.95, with 91 degrees of freedom, *p* ≤ 0.001. Intraclass correlation coefficient = 0.747 (0.697–0.793), with a 95% confidence interval, *F* value = 46.94, *p* ≤ 0.001.

The item reduction occurred specifically during the exploratory factor analysis (EFA). Items 2, 7, 9, 11, 14, 15, and 20 were excluded from the model because they exhibited low factor loadings (<0.40), high residual correlations, or cross-loadings that precluded a transparent and interpretable factor structure.

Table 5 shows significant correlations (*p* = 0.01) between the subtotal score for each of the four BDI-II factors, ranging from 0.240 to 0.341, and the total score, identifying correlations ranging from 0.497 to 0.740. This analysis revealed coherence and consistency within the instrument.

### 3.4. Confirmatory Factor Analysis

**Figure 2:**
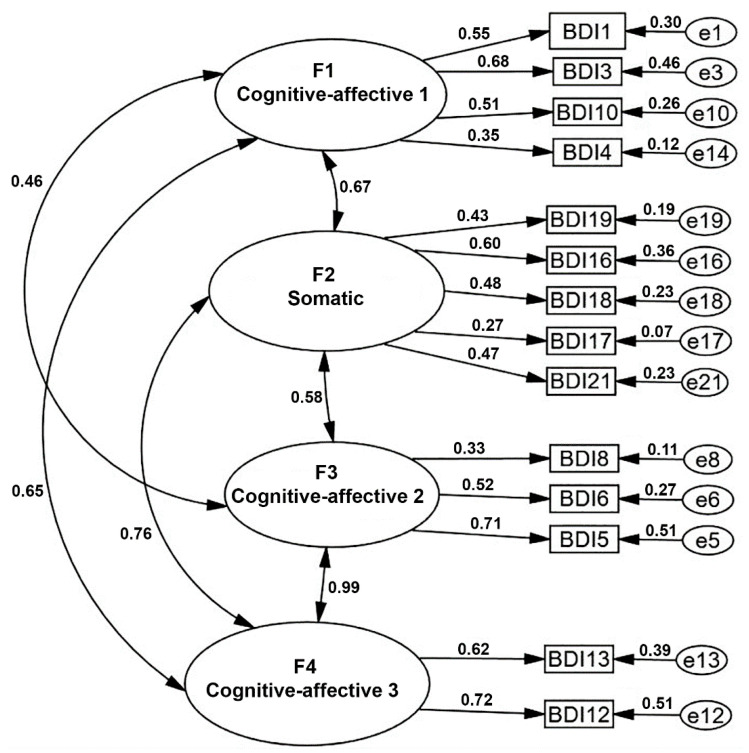
Confirmatory factor analysis (CFA) model for the BDI-II in the Otomi population of the Mezquital Valley. The analysis was performed on data from 138 participants. CMIN = 101.258; CMIN/DF = 1.426; GFI = 0.908; CFI = 0.901; TLI = 0.873; RMR = 0.055; PNFI = 0.581; RMSEA = 0.056 IC 90% [0.028–0.079]; Hoelter, n = 138, (*p* = 0.001).The squares with the text “BDI” followed by a number correspond to each item of the BDI questionnaire (same item numbering as in Table 4), and the circles with a letter “e” followed by a number represent the error in the explained variance for each item.

The first-order structural model that emerged from the exploratory analysis was confirmed using the maximum likelihood method, with four factors (Figure 2). The confirmatory factor analysis indicated that the model demonstrated good parsimony and acceptable fit to the observed data. The chi-square to degrees of freedom ratio (CMIN/DF = 1.43, *p* = 0.011) and the minimum discrepancy (CMIN = 101.26) supported the adequacy of the proposed structure. The Comparative Fit Index (CFI = 0.901) and the Goodness-of-Fit Index (GFI = 0.908) met the conventional thresholds (≥0.90), indicating a satisfactory model fit. The Root Mean Square Error of Approximation (RMSEA = 0.056; 95% CI [0.028–0.079]) showed a good fit of the model, reaffirmed by the value of the mean residual error (RMR = 0.055), which is clearly below the limit value (less than 0.06). The Tucker–Lewis index (TLI = 0.873) indicates a moderate fit of the proposed model. Although the value does not reach the recommended threshold of 0.90, the model is considered partially adequate and is interpreted in conjunction with other fit indices (RMSEA, CFI). Finally, the Hoelter test (n = 138, *p* = 0.01) indicated that the sample used is sufficient for the analysis.

Table 6 presents the results of the convergent and discriminant validity indices, based on the average variance extracted (AVE), the squared root of AVE, and composite reliability (CR).

## 4. Discussion

### 4.1. Main Contribution

The main contribution of this study was to develop and psychometrically evaluate a cross-cultural adaptation of the BDI-II for the Otomi indigenous population of the Mezquital Valley, demonstrating its potential use and ease of application. This adaptation addresses the need for culturally relevant instruments for specific groups, considering that most indigenous adaptations have been carried out in Africa, Australia, and Asia, and only 11.5% in Latin America and the Caribbean [49].

The use of non-adapted instruments can lead to inaccurate diagnostic interpretations and contribute to structural inequalities in mental health [50]. Although recent studies have validated the BDI-II in Indigenous populations of the northern plains, contributing to fairer and more culturally sensitive assessments [51], research of this type remains scarce, and the clinical and legal implications of using instruments without cross-cultural validation remain unknown. Cross-cultural adaptation is essential to ensure relevance, acceptability, and diagnostic accuracy in specific sociocultural contexts; it also promotes equity in mental health care [52].

In this sense, the results of the adaptation of the BDI-II for the Otomi population of the Mezquital Valley lay the foundation for integrating a more accurate assessment of depression into local health services, guaranteeing a reliable and valid measurement, as well as for the development of evidence-based prevention and treatment strategies. The identified factor structure reveals specific patterns of emotional distress within this population, facilitating more accurate screening.

### 4.2. Depression in the Otomi Population of the Mezquital Valley

The significant difference in depression compared with the absence of a chronic illness suggests a possible underestimation of the risk of depression in people without a previous medical diagnosis, or it could be related to greater contact with health services among those living with chronic illnesses. Psychosocial factors such as isolation, economic stress, or lack of support networks may negatively influence their mental health, independent of their physical condition [53]. Although previous studies have associated depression with chronic illnesses [54], exposure to inequalities in social determinants of health, such as poverty, food insecurity, and lack of access to health services, has also been found to be associated with an increased risk of depressive symptoms [55]. This finding suggests that even in the absence of diagnosed chronic illnesses, adverse social conditions may contribute to the development of depressive symptoms.

The median age of the participants with depression was higher than that of participants without depression: 54 (53–58) vs. 42 (40–49). This could be due to factors related to aging in this population, such as greater functional limitations, social isolation, and the cumulative burden of socioeconomic and cultural stress that impacts their mental health [56].

### 4.3. Factorial Structure of BDI-II in the Otomi Population of the Mezquital Valley

The 14-item version of the instrument, distributed across four factors, presents adequate reliability for assessing depression (Cronbach’s α of 0.756). However, these subscales had moderate alpha values (α = 0.505–0.633), warranting caution in interpretation; the items require refinement to improve internal consistency. The elimination of seven items could be due to cultural differences in understanding the items, or to lower correspondence with the way the Otomi population of the Mezquital Valley expresses and names their emotional experiences, which could generate interpretive ambiguity or low clinical relevance in this context. The low AVE and CR values could be due to the removal of these items.

The factorial structure is comparable to another study that evaluates the factorial structure of the BDI in the context of indigenous peoples [35]. Although exploratory and confirmatory factor analyses reveal four factors, these align with the original two-factor structure identified by Beck (1996) [36] and with the findings in the indigenous population of Ecuador, who also found a two-factor structure aligned with Beck’s [35].

Our findings differ from those of other authors, who have reported complex structures involving up to three factors in the general population [6,17,39] and clinical population [13]. Our findings suggest possible cultural particularities in the manifestation and understanding of depressive symptoms in the Otomi population of the Mezquital Valley, which should be studied from both interdisciplinary and multidisciplinary perspectives.

The resulting model from this study is a four-factor model, aligning with Beck’s (1996) findings [36]: cognitive–affective and somatic. Factors one, three, and four correspond to the cognitive–affective factor, and factor two to the somatic factor. Items seventeen and twenty-one, originally cognitive–affective, were grouped under the somatic factor, consistent with the three-factor model reported in clinical populations [37]. This finding suggests that, within the worldview of the Otomi indigenous population of the Mezquital Valley, depression is experienced and expressed predominantly through cognitive and somatic components, which coincides with Beck’s theory of the negative cognitive triad that impacts how we perceive ourselves, the world, and the future, manifesting through both cognitive and somatic symptoms [57].

The three factors corresponding to the cognitive–affective are distinguished from one another by the nature and direction of the depressive manifestations they encompass. Cognitive–affective factor 1 comprises a group of externalized symptoms visible to others, such as sadness, crying, feelings of failure, and loss of pleasure; cognitive–affective factor 2 encompasses emotionally internalized manifestations, such as self-criticism and feelings of guilt and punishment; while cognitive–affective factor 3 is characterized by attitudinal and behavioral expressions, evidenced by loss of interest and indecisiveness. These substructures reveal unique cultural nuances in the cognitive perception of depression, expanding Beck’s model by showing that its core components remain, but with specific manifestations that reflect the cultural logic of the Otomi population.

Previous research has confirmed this factorial structure, revealing two underlying factors that assess cognitive–affective and somatic symptoms of depression in the general Mexican population [19,38] and in the clinical population [20], showing that the items of the inventory reflect cognitive and somatic dimensions of depression, which supports both its clinical and psychometric usefulness.

The convergent validity of the BDI-II in the Otomi population was lower than the conventional criteria (AVE < 0.50; CR < 0.70) [58], indicating that the items only partially explain the variance of their factors. Similarly, discriminant validity was moderate, with some factors more differentiated than others. On the other hand, the authors of a cross-cultural validation of a well-being scale in the Kankuamo population of Colombia noted that the convergent/discriminant validity analyses were inconclusive [59]. This could be because it involves the manifestations and understanding of a construct—in our case, depressive symptoms. Therefore, careful adaptations and more research are needed to verify whether standardized instruments can be fully adapted to the indigenous context.

### 4.4. Cultural Contextualization of Depressive Symptoms

Although somatic symptoms have been reported to predominate in Mexican indigenous communities [60], our Otomi sample shows a greater cognitive–affective weight. This divergence suggests that depressive symptoms’ expression is not homogeneous among indigenous peoples and that symptomatic patterns may vary according to cultural context, instrument, and community characteristics, reinforcing the contribution of our findings by demonstrating a distinct manifestation of depression symptoms.

Several studies have documented that the conceptions of health and mental illness among Mexican indigenous peoples differ from the Western biomedical model and are structured within their own cultural frameworks. In Mayan communities, for example, mental health is conceived as an inseparable part of general health, in which biological, ontological, relational, and spiritual dimensions converge in a dynamic system that integrates traditional practices and biomedical resources adapted to their own perspectives [61]. On the other hand, indigenous youth in Oaxaca experience psychological suffering linked to social, ethnic, and migratory determinants [62]. In Latin American populations, it is common for psychological distress to be expressed through physical symptoms, a phenomenon related more to cultural and linguistic particularities than to an inherent ethnic condition [63].

Taken together, these results suggest that emotional distress in Indigenous contexts can be expressed in a culturally specific way. The predominance of cognitive–affective symptoms in the Otomi population does not constitute a deviation from the theoretical model, but rather a manifestation of the condition within their cultural framework. This indicates that instruments developed from Western perspectives may not fully capture the depressive experience in these groups, and that the differential functioning observed in some items is more related to cultural differences in the conceptualization and expression of suffering than to measurement flaws. Therefore, the need to interpret the results and adapt clinical instruments with intercultural sensitivity, considering the sociocultural context in which the symptoms emerge, is reinforced.

### 4.5. Potential Impact of the Cross-Culturally Adapted BDI-II

Adapting psychological instruments for Indigenous populations has proven feasible and relevant for ensuring measurement validity. In the Otomi population of the Mezquital Valley, the Center for Epidemiological Studies Depression Scale (CES-D) was previously adapted to the Ñahñú context (the endonym or self-designation of the Otomi people of the Mezquital Valley) to assess depression, although without confirmatory analysis [64]. In contrast, the present study provides additional evidence by adapting and validating the BDI-II in this context, reinforcing the importance of adapting tools to intercultural realities for culturally relevant and equitable assessments. The similarities and differences found, compared with previous research, suggest possible cultural particularities in the understanding and expression of depression that warrant exploration in future studies.

Content validity analysis provided strong evidence of the cultural and conceptual appropriateness of the Beck Depression Inventory-II (BDI-II) adapted for the Otomi population. This analysis has already been documented in the systematic application of CVI in cross-cultural adaptations of health instruments [65,66], and in specific instruments for indigenous people [67]. The results showed high levels of agreement and satisfactory content validity indices (CVI) across all dimensions. This confirms that the items adequately reflect the depressive construct and are understandable from a semantic and cultural perspective, supporting the instrument’s suitability for further psychometric evaluation. We agree with recent studies that demonstrate adaptation extends beyond mere translation and necessitates an evaluation of conceptual equivalence [32,68,69]. This process was implemented in our study through rigorous cross-cultural adaptation to ensure cultural appropriateness and semantic equivalence of the BDI-II items for the Otomi population.

### 4.6. Study Limitations

Due to the nature of the study, the results only reflect the behavior of the general population evaluated and cannot be directly extrapolated to clinical populations with severe symptoms. Furthermore, the sampling conducted in public spaces may have limited the participation of individuals with severe depressive symptoms. Another significant limitation is the lack of a test–retest analysis, which prevented the assessment of the instrument’s temporal stability due to geographical difficulties in obtaining a second measurement, and to the study design, which was based on a sample collected in a public park, limiting the possibility of evaluating temporal stability. Also, convergent validity was not assessed, limiting the evidence that the instrument consistently measures related constructs due to the lack of psychometric instruments.

Furthermore, although the total score showed acceptable internal consistency, several subscales had moderate alpha values (α = 0.505–0.633), suggesting caution in interpreting them. Regarding model fit, a TLI of 0.873 was obtained, slightly below the reference value of 0.90, which may reflect cultural differences in interpreting some items among the Otomi population. Finally, the removal of items during the exploratory factor analysis may have led to overfitting and low AVE and CR values, reinforcing the need for cautious interpretations and future validation in independent samples.

Future research could help evaluate the factor structure in larger, more heterogeneous independent samples, explore refinement alternatives to improve the instrument’s reliability, assess convergent and discriminant validity, consider longitudinal designs to evaluate the instrument’s temporal stability (test–retest), and perform a systematic face validity assessment with a larger number of members of the target population.

## 5. Conclusions

The BDI-II is a culturally relevant instrument with adequate validity and reliability for assessing depressive symptoms in the indigenous Otomi population of the Mezquital Valley. It facilitates the rigorous evaluation of these symptoms and overcomes the previous limitation stemming from the lack of psychometric tools adapted to this population. Although this study offers an initial approach to the instrument’s behavior in the Otomi population, its results should be interpreted with caution due to methodological limitations. The resulting factor structure aligns with Beck’s cognitive theory, supporting its conceptual validity for use in future studies and for patients’ assessment by mental health professionals working with this population.

## Figures and Tables

**Table 1:** Content validity analysis of BDI-II translated to Otomí of the Mezquital Valley.

Item	I-CVI						UA					
	R	W	L	T	A	C	R	W	L	T	A	C
1	1	1	1	1	1	1	1	1	1	1	1	1
2	1	1	1	0.7	1	1	1	1	1	0	1	1
3	0.7	1	1	1	1	1	0	1	1	1	1	1
4	1	1	1	1	1	1	1	1	1	1	1	1
5	1	1	0.7	1	1	1	1	1	0	1	1	1
6	1	0.7	0.7	1	1	1	1	0	0	1	1	1
7	1	1	1	1	1	1	1	1	1	1	1	1
8	1	1	1	1	1	1	1	1	1	1	1	1
9	1	0.7	1	1	1	1	1	0	1	1	1	1
10	1	1	1	1	1	1	1	1	1	1	1	1
11	1	1	1	1	1	1	1	1	1	1	1	1
12	1	0.7	1	1	1	1	1	0	1	1	1	1
13	1	0.7	1	1	1	1	1	0	1	1	1	1
14	1	1	1	1	1	0.7	1	1	1	1	1	0
15	1	1	1	1	1	1	1	1	1	1	1	1
16	1	1	1	1	1	1	1	1	1	1	1	1
17	1	1	1	1	1	1	1	1	1	1	1	1
18	1	1	1	1	1	1	1	1	1	1	1	1
19	1	1	1	1	1	1	1	1	1	1	1	1
20	1	1	1	1	1	1	1	1	1	1	1	1
21	0.7	1	1	1	1	1	0	1	1	1	1	1
S-CVI/Ave	0.97	0.94	0.97	0.98	1.00	0.98						
S-CVI/UA							0.90	0.81	0.90	0.95	1.00	0.95
Average proportion of items judged as relevant across the three experts	0.97	0.92	0.97	0.98	1.00	0.98						

Note: R = Relevance, W = writing, L = language appropriate for the population, T = theoretical validity, A = appearance validity, C = content validity, I-CVI = item-level content, UA = Universal agreement, validity index, S-CVI/Ave = scale-level content validity index based on the average method, S-CVI/UA = scale-level content validity index based on the universal agreement method.

**Table 2:** Proportion relevance of BDI-II per expert, calculated from all 21 items for each criterion.

Expert 1						Expert 2						Expert 3					
R	W	L	T	A	C	R	W	L	T	A	C	R	W	L	T	A	C
0.90	1	0.95	1	1	1	1	0.90	0.95	0.95	1	0.95	1	0.90	1	1	1	1

Note: R = Relevance, W = writing, L = language appropriate for the population, T = theoretical validity, A = appearance validity, C = content validity.

**Table 3:** Sociodemographic and clinical characteristics of the general Otomí population of the Mezquital Valley (n = 228). Data are presented as means and standard deviations, or absolute numbers (percentage).

Variable	Total Sample (n = 228)	Depression ≥9 Points n = 170 (75%)	No Depression ≤8 Points n = 58 (25%)	Test Statistic	gl	*F* (Levene)	Effect Size	*p*
Age	51 (41–60)	54 (53–58)	42 (40–49)	*U* = 3070.50	-	-	r = 0.28	≤0.001
Sex				*χ*^2^ = 2.79	1	-	Φ = 0.11	0.095
Female	139 (61%)	109 (64%)	30 (52%)					
Male	89 (39%)	61 (36%)	28 (48%)					
Level of education				*χ*^2^ = 5.34	4	-	Φ = 0.08	0.254
Without studies	35 (15%)	30 (18%)	5 (9%)					
Primary	74 (33%)	55 (32%)	19 (33%)					
Secondary	88 (38%)	63 (37%)	25 (43%)					
Baccalaureate	20 (9%)	16 (9%)	4 (7%)					
Technician or professional	11 (5%)	6(4%)	5 (9%)					
Marital status				*χ*^2^ = 3.44	4	-	Φ = 0.06	0.486
Single	25 (11%)	20 (12%)	5 (9%)					
Married	101 (44%)	74 (43%)	27 (47%)					
Free Union	83 (36%)	59 (35%)	24 (41%)					
Widower	16 (7%)	14 (8%)	2 (3%)					
Divorced	3 (1%)	3 (2%)	0 (0%)					
Chronic illness				*χ*^2^ = 4.03	1	-	Φ = 0.13	0.045
Yes	84 (37%)	69 (41%)	15 (26%)					
No	144 (63%)	101 (59%)	43 (74%)					
Health Insurance				*χ*^2^ = 0.086	1	-	Φ = 0.02	0.769
No	202 (89%)	150 (88%)	52 (90%)					
Yes	26 (11%)	20 (12%)	6 (10%)					
Psychological care				*χ*^2^ = 0.29	1	-	Φ = 0.01	0.865
No	195 (85%)	145 (85%)	50 (86%)					
Yes	33 (15%)	25 (15%)	8 (14%)					
Symptoms of depression (points)	14.3 ± 8.4	21.5 ± 6.5	8.05 ± 3.5	*t* = −20.04	224.30	52.06	d = 2.68	≤0.001
Cognitive–affective 1	3.6 ± 2.5	4.4 ± 2.3	1.2 ± 1.2	*t* = −13.67	183.71	16.72	d = 2.02	≤0.001
Somatic	4.0 ± 3.1	4.9 ± 3.0	1.6 ± 1.6	*t* = −10.58	186.98	25.61	d = 1.55	≤0.001
Cognitive–affective 2	1.5 ± 1.6	1.8 ± 1.7	0.7 ± 0.9	*t* = −6.47	190.94	27.22	d = 0.94	≤0.001
Cognitive–affective 3	0.8 ± 1.3	1.0 ± 1.4	0.1 ± 0.4	*t* = −7.76	216.39	85.02	d = 1.05	≤0.001

Note. Data are presented as median or mean ± standard deviation, as appropriate. *U* = Mann–Whitney U test; *χ*^2^ = chi-square test; *t* = Student’s *t*-test; r = effect size for U; Φ = effect size for *χ*^2^; d = Cohen’s d effect size. A significance level of *p* < 0.05 was set.

**Table 4:** Exploratory 4-factor structure of the Beck Depression Inventory-II (BDI-II), *n* = 228.

Factor/Item	λ	x‾	SD	α	x‾	SD	σ^2^	ICC	ICC L	ICC H	Hotelling F	*p*
Factor 1. Cognitive–affective 1				0.627	3.62	2.48	6.11	0.628	0.543	0.701	72.55	≤0.001
1. Sadness	0.795	1.03	0.96									
3. Failure	0.615	0.72	0.88									
10. Crying	0.612	1.43	0.94									
4. Loss of Pleasure	0.533	0.44	0.78									
F2. Somatic				0.604	4.02	3.05	9.29	0.589	0.498	0.667	50.86	≤0.001
19. Difficulty Concentrating	0.700	0.48	0.73									
16. Changes in Sleep Habits	0.635	1.03	1.17									
18. Changes in appetite	0.596	0.54	0.88									
17. Irritability	0.552	1.39	1.11									
21. Loss of Interest in Sex	0.497	0.59	0.99									
F3. Cognitive–affective 2				0.505	1.49	1.59	2.54	0.505	0.383	0.607	14.87	≤0.001
8. Self-criticism	0.708	0.61	0.70									
6. Feelings of Punishment	0.651	0.55	0.86									
5. Feelings of Guilt	0.581	0.32	0.66									
F4. Cognitive–affective 3				0.633	0.79	1.29	1.67	0.6	0.481	0.692	29.54	≤0.001
13. Indecision	0.774	0.54	0.89									
12. Loss of interest	0.644	0.25	0.60									

Note. λ = factor loading; x‾ = item mean; SD = standard deviation; α = Cronbach’s alpha; x‾ = factor mean; σ^2^ = variance; ICC = intraclass correlation coefficient; ICC L = lower limit of the ICC; ICC H = upper limit of the ICC; Hotelling F = Hotelling’s T^2^ *F*-value; *p* = significance level.

**Table 5:** Spearman correlation between BDI-II factors (BDI-II), *n* = 228.

Factors	F1	F2	F3	F4
F1. Cognitive–affective 1	1			
F2. Somatic	0.341 **	1		
F3. Cognitive–affective 2	0.252 **	0.240 **	1	
F4. Cognitive–affective 3	0.318 **	0.300 **	0.291 **	1
Total depression score	0.740 **	0.738 **	0.497 **	0.557 **

Note. Spearman correlation coefficients are presented. F1–F4 correspond to the four factors of the scale: F1 = Cognitive–affective 1, F2 = Somatic, F3 = Cognitive–affective 2, F4 = Cognitive–affective 3. The total depression score represents the sum of all items. ** indicates correlations significant at *p* < 0.01. All tests are two-tailed.

**Table 6:** Convergent and discriminant validity of the BDI-II factors in the Otomi population (n = 138).

Factor	AVE	$\sqrt{AVE}$	CR
1	0.29	0.53	0.60
2	0.22	0.47	0.57
3	0.30	0.54	0.54
4	0.45	0.67	0.62

Note. AVE = Average Variance Extracted; CR = Composite Reliability; $\sqrt{AVE}$ = square root of AVE; values below 0.50 (AVE) and 0.70 (CR) indicate limited convergent validity.

## Data Availability

The authors will make the raw data supporting the conclusions of this article available on request. The data are not publicly available due to institutional policies.

## Abbreviations

The following abbreviations are used in this manuscript:

ICC	Correlation Coefficient
CFI	Comparative Fit Index
RMSEA	Root Mean Square Error of Approximation
GFI	Goodness-of-Fit Index
INEGI	National Institute of Statistics and Geography
CFA	Confirmatory Factor Analysis
